# Information Technology-Based Intervention on the Socio-Emotional Competence of Individuals with Autism Spectrum Disorders: A Systematic Review and Meta-Analysis

**DOI:** 10.3390/jintelligence13080098

**Published:** 2025-08-04

**Authors:** Yunshan Liu, Sirao Li, Yaping Huang, Dan Li

**Affiliations:** School of Education, Hunan Normal University, Changsha 410081, China; 202230015014@hunnu.edu.cn (Y.L.); fancy@hunnu.edu.cn (S.L.);

**Keywords:** autism spectrum disorders, information technology, social emotion competence, meta-analysis, systematic review

## Abstract

Individuals with autism spectrum disorder (ASD) have deficits in social–emotional competence. Most people with ASD have difficulties in emotion recognition, emotion understanding, emotion expression, and emotion regulation, which seriously affects their normal social communication and interaction. The information technology (IT) era has given more possibilities for intervention training for people with ASD, and research has proven that technological interventions have a significant effect on the socio-emotional competence of people with ASD. This study employed a meta-analytic approach using 32 independent effect sizes from 25 studies to investigate the effects of IT interventions on socio-emotional competence in individuals with ASD, using emotion recognition, understanding, expression, and regulation as dependent variables and examining key moderating factors. The results found that information technology has an excellent effect on social–emotional competence in ASD (Hedges’ g = 0.897, CI = 0.676, 1.117, z = 7.967, *p* < 0.001) and is significantly moderated by the intervention technique (Q = 7.392, *p* = 0.025) and the intervener (Q = 4.933, *p* = 0.026). The findings provide insights into further deepening information technology intervention research as well as practical applications.

## 1. Introduction

Autism spectrum disorder (ASD) is a lifelong neurodevelopmental condition characterized by pervasive challenges in social communication and interaction, alongside restricted and repetitive patterns of behavior, interests, or activities (American Psychiatric Association 2013). Among the most debilitating aspects of ASD are deficits in socio-emotional competence—a multidimensional construct encompassing emotion recognition (identifying emotional cues in others), emotion understanding (interpreting the context and meaning of emotions), emotion expression (conveying one’s own emotions appropriately), and emotion regulation (managing emotional responses adaptively) (Lievore et al. 2023). These impairments often manifest as difficulties in interpreting facial expressions, understanding implicit social norms, responding empathetically, or modulating emotional reactions in dynamic social environments (Capps et al. 2009; Pouw et al. 2013). Such challenges not only impede the formation of meaningful relationships but also contribute to social isolation, academic underachievement, and comorbid mental health conditions such as anxiety and depression (Bruggink et al. 2015; Reyes et al. 2020). Consequently, fostering socio-emotional competence has emerged as a critical target for interventions aimed at improving functional outcomes and quality of life for individuals with ASD.

Traditional therapeutic approaches, including cognitive–behavioral therapy (CBT), play-based interventions, and social skills training, have demonstrated modest efficacy in addressing socio-emotional deficits (Weiss et al. 2018; Williams et al. 2012). However, their real-world applicability is often constrained by logistical barriers, such as reliance on highly trained professionals, limited accessibility in low-resource settings, and difficulties in maintaining engagement among individuals with ASD, particularly those with sensory sensitivities or limited verbal abilities (Grynszpan et al. 2013; Thomeer et al. 2015). Moreover, conventional methods frequently struggle to replicate the complexity and unpredictability of real-life social interactions, limiting the generalization of learned skills to naturalistic contexts (Didehbani et al. 2016).

The proliferation of information technology (IT) offers a transformative opportunity to overcome these limitations. IT-based interventions—ranging from virtual reality (VR) simulations and augmented reality (AR) applications to robotic systems and gamified mobile platforms—leverage the inherent strengths of individuals with ASD, such as heightened visual processing and affinity for structured, predictable environments (Lorenzo et al. 2016; Marino et al. 2019). For instance, VR systems immerse users in customizable, controlled social scenarios, enabling the repetitive practice of emotion recognition without the sensory overload typical of face-to-face interactions (Ip et al. 2018). Similarly, social robots like NAO or Pepper provide consistent, nonjudgmental interaction partners, facilitating emotion regulation through predictable, rule-based exchanges (Holeva et al. 2024). Gamified applications, such as Emotiplay or Mind Reading, employ interactive narratives and immediate feedback to reinforce emotional understanding and expression (Fridenson-Hayo et al. 2017; Golan and Baron-Cohen 2006). Crucially, these tools can be tailored to individual needs, scaled for remote delivery, and integrated into daily routines, thereby enhancing accessibility and adherence (Fage et al. 2019; Lyu et al. 2023).

Despite growing enthusiasm for IT-based solutions, empirical evidence remains fragmented and contradictory. While some studies report robust improvements in emotion recognition following VR training (Didehbani et al. 2016) or tablet-based programs (Golan et al. 2010), others observe limited generalization or transient effects (Williams et al. 2012). Discrepancies also persist regarding the role of moderating variables, such as intervention duration, technological modality, and the expertise of implementers. For example, desktop-based interventions (e.g., video modeling) have shown superior efficacy in emotion recognition compared to mobile apps (Zhi et al. 2021), while interventions delivered by trained professionals yield significantly better outcomes than those administered by caregivers (Salvador et al. 2015). Furthermore, the differential impacts of IT tools across distinct socio-emotional domains—such as emotion regulation versus expression—remain underexplored, with most studies focusing narrowly on isolated skills (Rashidan et al. 2021; Valentine et al. 2020).

To address these gaps, this systematic review and meta-analysis synthesizes evidence from randomized controlled trials (RCTs) and quasi-experimental studies to achieve three objectives: (1) Quantify the overall efficacy of IT-based interventions on the four core dimensions of socio-emotional competence (recognition, understanding, expression, regulation) in individuals with ASD. (2) Identify moderating factors influencing intervention outcomes, including technological modality (e.g., VR, robotics, desktop/mobile tools), intervention duration/frequency, setting (structured vs. unstructured), and implementer role (professional vs. caregiver). (3) Provide evidence-based recommendations for optimizing the design, delivery, and scalability of future IT interventions in clinical and educational contexts.

By integrating findings across heterogeneous methodologies and populations, this study not only clarifies the current state of the field but also highlights pathways for bridging the gap between technological innovation and practical implementation.

## 2. Methods

### 2.1. Registration

This review is registered in the International Prospective Register of Systematic Reviews (PROSPERO) (CRD 42024554006). According to the guidelines outlined in the Preferred Reporting Items for Systematic Reviews and Meta-Analyses (PRISMA-NMA) statement (Hutton et al. 2015), this review conducted a comprehensive search of the relevant literature.

### 2.2. Search Strategy

The Chinese databases (CNKI, VIP Database, WanFang Database) and English databases (Web of Science, Springer Link, Pub Med, IEEE) were searched, and the keywords for the search “autism, autism spectrum disorder/Asperger’s/autistic”, “technology”, (intervention/therapy/activities/therapeutic), “emotional recognition/e-motional understanding/emotional expression/emotional regulation” were searched in combination. The search time period was from January 2005 to May 2024. A supplementary search was also conducted for references to review papers related to the research topic. All the literature was managed by the Endnote 21 literature management software.

### 2.3. Study Inclusion and Exclusion Criteria

**Figure 1:**
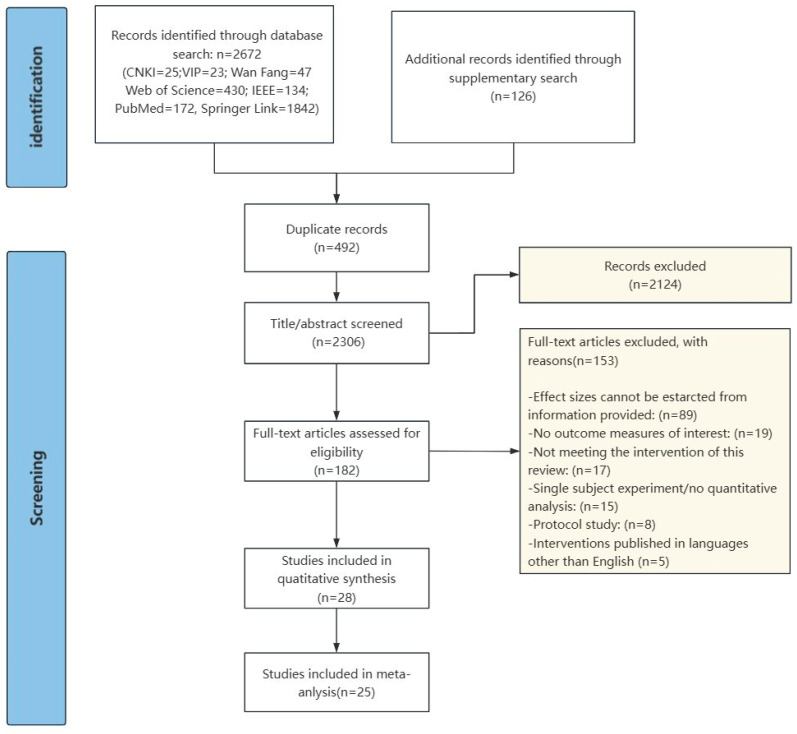
Flow chart of the literature screening.

The criteria for inclusion in the literature were (1) the study population had to include people with ASD; (2) the topic of the study was an empirical study of information technology interventions for social–emotional competence in ASD, including only randomized controlled experiments or quasi-experimental research methods; (3) peer-reviewed journal articles; and (4) empirical studies in which the articles could provide the sample sizes, the means and standard deviations of the scores on the relevant tests, and data that could be transformed into effect sizes of the data. Exclusion criteria were (1) duplicated literature; (2) literature with titles and abstracts that were clearly unrelated to the topic; (3) non-empirical studies; (4) studies with a single-subject experimental design; (5) literature with incomplete data or no data that could be converted into effect size indicators; and (6) non-journal articles and articles that were not peer-reviewed. Twenty-five articles were finally included in the meta-analysis, all of which were in English. The literature screening process is shown in Figure 1.

## 3. Data Extraction

In accordance with the nine dimensions of the literature coding proposed by H. Cooper (basic information about the study, study design, participants, study methodology, experimental treatment, measures of interest, effects, confidence scores, and iterative refinement) (Cooper et al. 2019), and taking into account the purpose of the study and the content of the study, data were extracted and coded in terms of the first author, time of publication, intervention goal, level of functioning, age, frequency of the intervention, sample size, interviewees, intervention duration, intervention technique, and intervention context. Eleven aspects of the literature data were extracted and coded as detailed in Supplementary Table S1. All data and literature coding were conducted independently by two researchers using identical standardized Excel extraction templates, followed by inter-rater reliability checks to ensure methodological rigor. Subject characteristics included the ASD functional level and age; intervention characteristics included sample size, intervener, intervention duration, intervention technique, and intervention context. In this study, intervener was categorized as researcher (experimentalist, rehabilitator, helper, etc.) and non-researcher (parent or teacher); intervention duration was categorized as 6 weeks and below or 6 weeks and above; IT tools were divided into mobile phones and wearables (all mobile devices that can be worn as personal items to access ICT-related functions, such as tablets, smartphones, smartwatches, etc.), desktop computers (videos, personal computers, and web-based technology), and emerging technologies (technological innovations in a particular field, including virtual reality, augmented reality, and robotics, etc.); and intervention contexts are divided into structured contexts (targeted training venues, including treatment center, training rooms, etc.) and unstructured contexts (home, school).

## 4. Quality Appraisal

Two researchers used the Risk of Bias 2 (RoB 2) tool developed by Cochrane (Sterne et al. 2016) to independently evaluate controlled trials for each study in five main areas: randomization process, deviation from intended intervention, missing outcome data, outcome measurement, and selection of the reported result. Uncontrolled experiments were primarily evaluated using the Risk Of Bias In Non-Randomized Studies—of Interventions (ROBINS-1) (Sterne et al. 2016). The tool consists of seven main areas of evaluation: pre-intervention (bias due to confounding, bias in the selection of participants into the study); at intervention (bias in the selection of participants into the study; bias in the classification of interventions); post-intervention (bias due to deviations from intended interventions, bias due to missing data, bias in the measurement of outcomes, bias in the selection of the reported result). The categories for risk of bias judgements are “Low risk”, “Moderate risk”, “Serious risk”, and “Critical risk” of bias. Any disagreements in the assessment results were judged by a third researcher.

## 5. Data Analysis and Synthesis

The literature data were mainly processed using Comprehensive Meta Analysis Version 3.0 (CMA3.0). As the data reported in all the studies included in this study were continuous variables, the experimental sample size, before-and-after means, and between-group *t*-values were mainly selected to calculate the effect sizes, and the standardized mean difference, Hedges’ g, was used as an indicator of the effect sizes. According to J. Cohen’s definition of effect size, effect sizes of 0.2, 0.5, and 0.8 correspond to the critical values of small, medium, and large effect sizes, respectively. Among them, controlled trials were mainly calculated using the sample size of the two groups, the post-test mean, and the t-value between the groups; uncontrolled trials, the sample size, and the *t*-value were mainly used to calculate. Considering the high heterogeneity of the results of different studies with large differences in sample sizes, outcome target measurement tools, etc., this review uses a random-effects model for meta-analysis. The I^2^ test will be used to assess the heterogeneity, with 25%, 50%, and 75% corresponding to low, medium, and high heterogeneity, respectively (Huedo-Medina et al. 2006). Based on the division of socio-emotional competence, the meta-analytic findings were categorized into four dimensions; (i) emotion recognition, (ii) emotion comprehension, (iii) emotion expression, and (iiii) emotion regulation. In addition, subgroup analyses were conducted in this study in an attempt to explore the potential impact of intervention characteristics on the findings.

### Social Validity

Based on the above criteria, the coding of the literature was performed independently by the two researchers, and the coding consistency was 94.32%. In the case of coding inconsistencies, the two researchers reviewed the original documents together and discussed and finally reached agreement.

## 6. Results

### 6.1. Study Characteristics

This review briefly extracted the features of the 28 included papers, and the extraction results are presented in Supplementary Table S1.

Study design: Of the 28 pieces of the literature included, there were 23 randomized controlled trials (RCTs) (RCTs = 22, controlled trials = 1), 4 single-group pre- and post-test trials and 1 quasi-experiment. A total of 1226 participants were involved in these 28 papers, of which 1140 were involved in the control, 76 in the single-group pre- and post-test experiments, and 20 in the quasi-experiment.

Functional level: Of the thirty-one studies that produced results, two studies did not report participants’ level of functioning, two studies had participants with low levels of functioning, and the remaining studies had participants with high levels of functioning. The age of the participants ranged from 4 to 30 years. There were 25 studies with a mean age of less than 12 years and 6 studies with participants aged 12 years and older.

Intervention categories: Thirteen studies resulted in the use of handset and wearables, twelve studies resulted in the use of emerging technology, and six studies resulted in the use of desktop computers. Of all the technological tools used, handset and wearables had the largest share of gaming software. Emerging technology was mainly virtual reality (*n* = 9) and robot-assisted interventions (*n* = 3).

Intervention duration: Overall, it ranged from 1 h to 24 weeks. In total, 12 of the 31 studies’ results showed intervention durations of 6 weeks or less, 17 had intervention durations of more than 6 weeks, and 2 studies did not report intervention durations.

Intervention frequency: Three studies reported the frequency of intervention; one study intervened a total of ten times; two studies intervened a total of ten hours; and one study’s intervention frequency indicated at least three episodes a day. Of the remaining findings, 3 studies had an intervention frequency of five or more; 24 had an intervention frequency of less than five.

Intervention Context: Of the 31 study results, 7 studies did not report the context of the intervention. Of the remaining 24 studies, 15 studies were primarily in unstructured contexts such as home and school; 9 studies occurred in structured contexts such as laboratories and equipped rooms. Of all the findings, three studies did not report the implementer of the intervention, four studies had interveners who were both peers and teachers familiar to the subjects and unfamiliar to the researchers, 1 study had no interveners and was conducted by the subjects alone; 8 studies were conducted by parents and teachers; and 15 studies were conducted by trained people such as research assistants, researchers, and therapists.

### 6.2. Quality Appraisal Results

Of the 28 studies included, this review assessed quality individually for each study. RCTs *(n* = 22) and QEDs (*n* = 1) were assessed using ROB2 developed by Cochrane, and single-group pre- and post-test trials were assessed using ROBINS-1 (*n* = 5). Specific assessments are shown in Table 1 and Table 2. In total, three studies were of low research quality and fifteen studies were ultimately included in the meta-analysis.

### 6.3. Meta-Analysis Results

A total of 25 studies were included in the meta-analysis, yielding a total of 32 findings. Of these, the effectiveness of IT interventions for emotion recognition (*n* = 20), emotion comprehension (*n* = 3), emotion expression (*n* = 5), and emotion regulation (*n* = 4) was reported. Due to the high degree of heterogeneity found in the characteristics of the included studies, random-effects meta-analyses were conducted to report overall effect sizes. Subgroup analyses were also conducted to examine the moderating effects of the studies and intervention characteristics.

**Figure 2:**
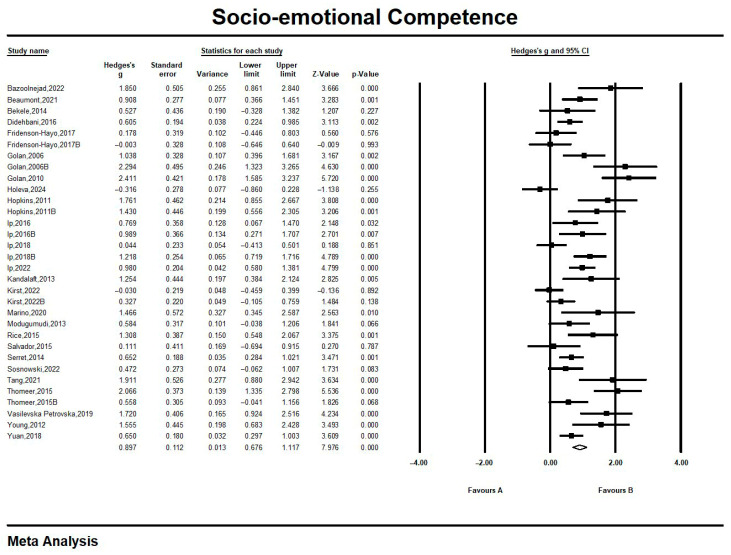
Effects of technical intervention on social–emotional competence. All references in the Figure 2: Bazoolnejad et al. (2022); Beaumont et al. (2021); Bekele et al. (2014); Didehbani et al. (2016); Fage et al. (2019); Fridenson-Hayo et al. (2017); Fridenson-Hayo et al. (2017); Golan and Baron-Cohen (2006); Golan and Baron-Cohen (2006); Golan et al. (2010); Herrero and Lorenzo (2020); Holeva et al. (2024); Hopkins et al. (2011); Ip et al. (2016); Ip et al. (2018); Ip et al. (2022); Kandalaft et al. (2013); Kirst et al. (2022); Lin et al. (2023); Marino et al. (2019); Modugumudi et al. (2013); Rice et al. (2015); Salvador et al. (2015); Serret et al. (2014); Sosnowski et al. (2022); Tang et al. (2021); Thomeer et al. (2015); Vasilevska Petrovska and Trajkovski (2019); Young and Posselt (2012); Yuan and Ip (2018). Effect sizes marked with B indicate that they are derived from the same article; similarly for those below.

As shown in Figure 2, the overall effect size of this study was 0.897, which is a large effect. This indicates that the information technology tool has achieved a high level of intervention effect on the socio-emotional competence of people with ASD, producing an excellent intervention effect.

#### 6.3.1. Emotion Recognition

**Figure 3:**
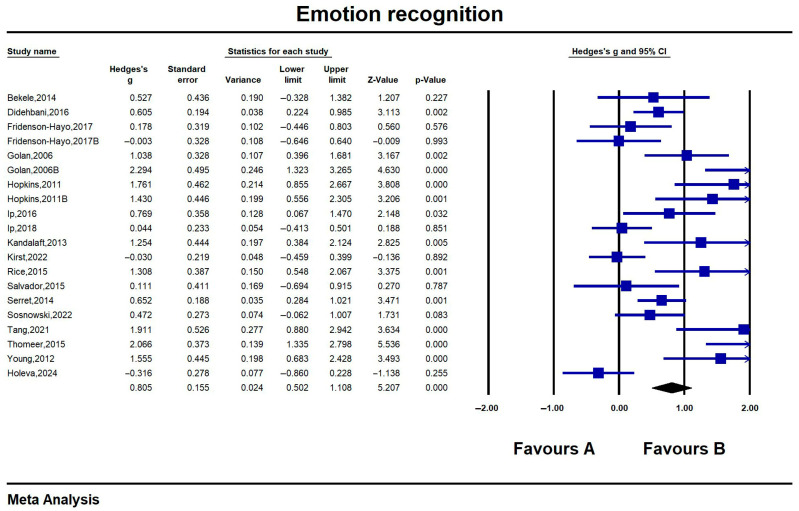
Forest plot-1 of emotion recognition. All references in the Figure 3: Bekele et al. (2014); Didehbani et al. (2016); Fridenson-Hayo et al. (2017); Fridenson-Hayo et al. (2017); Golan and Baron-Cohen (2006); Golan and Baron-Cohen (2006); Hopkins et al. (2011); Ip et al. (2016); Ip et al. (2018); Kandalaft et al. (2013); Kirst et al. (2022); Rice et al. (2015); Salvador et al. (2015); Serret et al. (2014); Sosnowski et al. (2022); Tang et al. (2021); Thomeer et al. (2015); Young and Posselt (2012); Holeva et al. (2024).

The results of the 20 combined studies on emotion recognition skills produced effect values ranging from −0.003 (−0.646, 0.640) to 2.066 (1.335, 2.798), with 8 studies having a non-significant intervention effect (*p* > 0.05), and a significant large effect was achieved for overall emotion recognition skills (Hedges’ g = 0.805, CI = 0.502, 1.108, z = 5.207, *p* < 0.001; I^2^ = 78.15%). The results showed that emotion recognition was significantly improved in people with ASD who received the IT intervention compared to the control group. Detailed results are shown in Figure 3.

#### 6.3.2. Emotion Understanding

**Figure 4:**
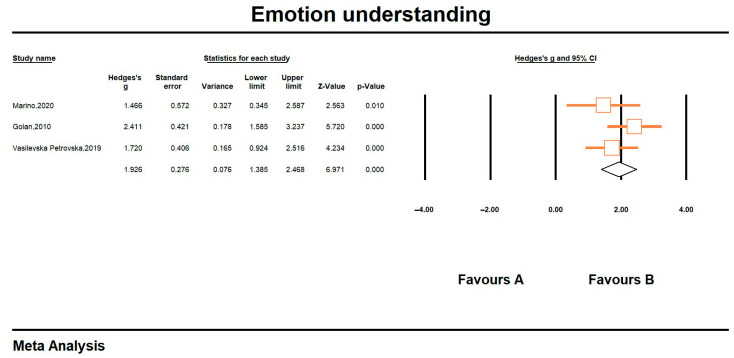
Forest plot-2 of emotion understanding. All references in the Figure 4: Marino et al. (2019); Golan et al. (2010); Vasilevska Petrovska and Trajkovski (2019).

Of the three studies on emotional comprehension, which produced effect values ranging from 1.466 (0.345, 2.587) to 2.411 (1.585, 3.237), there was a significant intervention effect in all of them (*p* < 0.05), with overall emotional comprehension reaching a significant large effect (Hedges’ g = 1.926, CI = 1.385, 2.468, z = 6.971, *p* < 0.001; I^2^ = 10.23%). The results showed that the emotional comprehension of people with ASD who received the IT intervention was significantly improved compared to the control group. Detailed results are shown in Figure 4.

#### 6.3.3. Emotion Expression

**Figure 5:**
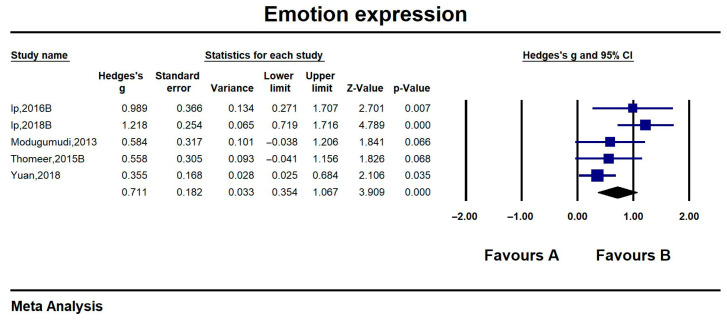
Forest plot-3 of emotion expression. All references in the Figure 5: Ip et al. (2016); Ip et al. (2018); Modugumudi et al. (2013); Thomeer et al. (2015); Yuan and Ip (2018).

Of the five studies on emotional expressiveness that produced effect values ranging from 0.355 (0.025, 0.684) to 1.218 (0.719, 1.716), two of the studies had a non-significant intervention effect (*p* > 0.05), and overall emotional comprehension reached a significant large effect (Hedges’ g = 0.711, CI = 0.354, 1.067, z = 3.909, *p* < 0.001; I^2^ = 55.81%). The results showed that the emotional comprehension of people with ASD who received the IT intervention was somewhat improved compared to the control group. Detailed results are shown in Figure 5.

#### 6.3.4. Emotion Regulation

**Figure 6:**
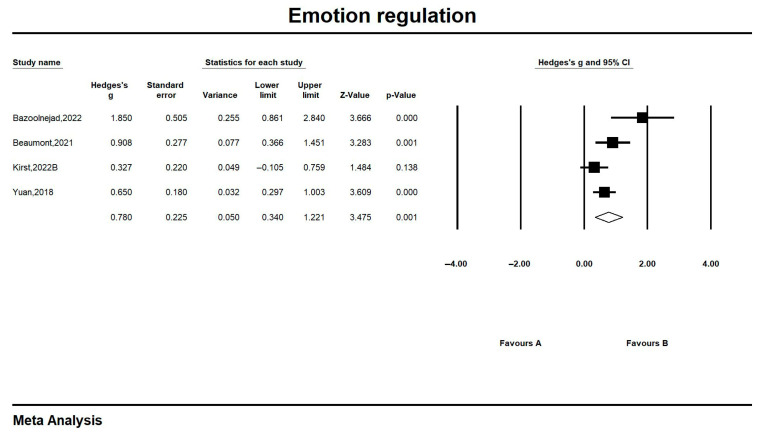
Forest plot-4 of emotion regulation. All references in the Figure 6: Bazoolnejad et al. (2022); Beaumont et al. (2021); Kirst et al. (2022); Yuan and Ip (2018).

Of the four studies on emotion regulation skills that produced effect sizes ranging from 0.327 (−0.105, 0.759) to 1.850 (0.861, 2.840), one study had a non-significant effect of the intervention (*p* > 0.05), and overall emotional comprehension skills reached a significant large effect (Hedges’ g = 0.780, CI = 0.340, 1.221, z = 3.475, *p* = 0.001; I^2^ = 65.31%). The results showed that the emotional comprehension of people with ASD who received the IT intervention was significantly improved compared to the control group. Detailed results are shown in Figure 6.

#### 6.3.5. Heterogeneity

The results of the meta-analysis on emotion recognition (Q = 86.942, df = 19, *p* = 0.000, I^2^ = 78.15%) of socio-emotional competencies in ASD as analyzed by the CMA software showed that there may be a high level of heterogeneity in the included studies and they may not share a common effect size. Furthermore, the high heterogeneity also suggests that there may be moderating factors that have an impact on the intervention effect. In contrast, the relatively low heterogeneity in the competencies of emotion comprehension (Q = 2.228, df = 2, *p* = 0.328, I^2^ = 10.23%), emotion expression (Q = 9.053, df = 4, *p* = 0.060, I^2^ = 55.81%), and emotion regulation (Q = 8.647, df = 3, *p* = 0.034, I^2^ = 65.306%) may indicate that the included studies for these two competencies will not share much similarity in terms of the characteristics of the intervention population, study characteristics, etc.

#### 6.3.6. Publication Bias and Influence on Findings

Publication bias was judged by a funnel plot and Egger’s test. The funnel plots of the four emotional competencies of social–emotional competence are shown in detail in Supplementary Figures S1–S4. From the funnel plots, we can find that there is a certain degree of bias in the distribution of the effect value of emotional recognition competence, indicating that there may be a publication bias, whereas the funnel plots of the rest of the emotional competencies as a whole show a symmetrical distribution, with a smaller possibility of publication bias. For further confirmation, we analyzed the data using Egger’s test. For emotion recognition ability, Egger’s test results indicated (t = 3.123, df = 18, *p* = 0.006) the presence of publication bias. For emotional comprehension ability, Egger’s test results indicated (t = 0.616, df = 1, *p* = 0.648) that there was no publication bias. For emotional expressiveness, Egger’s test results indicated (t = 1.172, df = 3, *p* = 0.326), suggesting no publication bias. For emotion regulation, Egger’s test results indicated (t = 1.971, df = 2, *p* = 0.187) that there was no publication bias.

#### 6.3.7. Subgroup Analyses

In addition to a meta-analysis of socio-social–emotional competence, this review conducted subgroup analyses to explore the moderating effects of intervention technology, intervention duration, intervention frequency, intervention context, and intervener’s presence in the intervention characteristics of the study outcomes.

**Figure 7:**
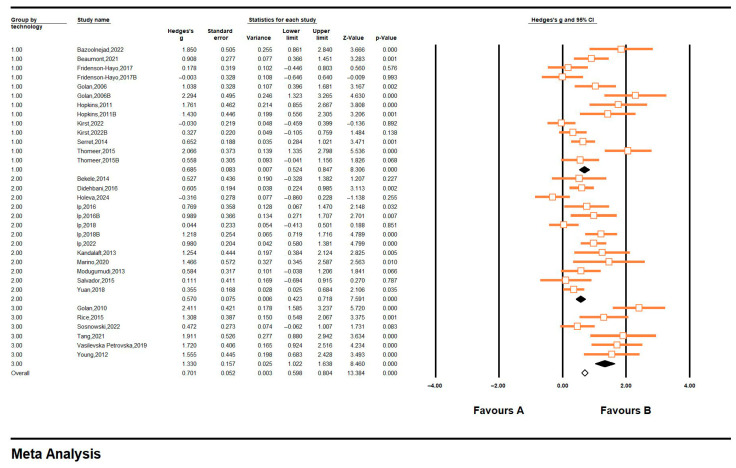
Forest plot-5 of technology categories 1, 2, and 3 represent mobile phones and wearables, emerging technologies, and desktop computers. All references in the Figure 2: Bazoolnejad et al. (2022); Beaumont et al. (2021); Bekele et al. (2014); Didehbani et al. (2016); Fage et al. (2019); Fridenson-Hayo et al. (2017); Fridenson-Hayo et al. (2017); Golan and Baron-Cohen (2006); Golan and Baron-Cohen (2006); Golan et al. (2010); Herrero and Lorenzo (2020); Holeva et al. (2024); Hopkins et al. (2011); Ip et al. (2016); Ip et al. (2018); Ip et al. (2022); Kandalaft et al. (2013); Kirst et al. (2022); Lin et al. (2023); Marino et al. (2019); Modugumudi et al. (2013); Rice et al. (2015); Salvador et al. (2015); Serret et al. (2014); Sosnowski et al. (2022); Tang et al. (2021); Thomeer et al. (2015); Vasilevska Petrovska and Trajkovski (2019); Young and Posselt (2012); Yuan and Ip (2018).

In terms of the intervention technology category, mobile phones and wearables (N = 13, Hedges’ g = 0.916, CI = 0.537, 1.294), emerging technologies (N = 13, Hedges’ g = 0.611, CI = 0.342, 0.881), and desktop computers (N = 6, Hedges’ g = 1.517, CI = 0.898, 2.136) all produced moderate and above effect sizes with positive intervention effects. The implementation effects of different technological interventions exhibit significant differences (Q = 7.392, df = 2, *p* = 0.025), and the desktop computers perform the best intervention, suggesting that the significant effect sizes for these three outcomes may be predicted by the means of intervention included in this study. See Figure 7 for details.

In terms of intervention duration, the length of the study implementation produced large effects at 6 weeks and less (N = 14, Hedges’ g = 1.018, CI = 0.658, 1.379) and 6 weeks and more (N = 16, Hedges’ g = 0.872, CI = 0.544,1.199), indicating that all had highly significant intervention effects. However, whether or not the length of the intervention exceeded 6 weeks did not produce a significant between-group difference (Q = 0.349, df = 1, *p* = 0.555). This indicates that there was no significant moderating effect of intervention duration on the intervention effect. For details, see Supplementary Figure S5.

In terms of intervention frequency, implementing an IT-based tool produced large effects for interventions twice a week and below (N = 21, Hedges’ g = 0.965, CI = 0.685, 1.245), and twice a week and above (N = 7, Hedges’ g = 0.986, CI = 0.398,1.573) all produced large effects, suggesting that all had highly significant intervention effects. However, intervention frequency did not produce a significant difference between groups (Q = 0.004, df = 1, *p* = 0.951). This indicates that there was no significant moderating effect of intervention frequency on the intervention effect. For details, see Supplementary Figure S6.

In terms of intervention contexts, the implementation of IT-based tools in structured contexts (N = 16, Hedges’ g = 0.947, CI = 0.609, 1.286) and unstructured contexts (N = 7, Hedges’ g = 0.958, CI = 0.510, 1.405) all produced large effects, indicating that all had highly significant intervention effects. However, studies conducted in different intervention contexts did not produce significant differences (Q = 0.001, df = 1, *p* = 0.971). This indicates that there was no significant moderating effect of the intervention context on the intervention effect. For details, see Supplementary Figure S7.

In terms of interveners, the technological means to intervene in social–emotional competence was moderated by parents and teachers (N = 8, Hedges’ g = 0.562, CI = 0.154, 0.970), researchers (therapists, assistants, etc.) (N = 18, Hedges’ g = 1.137, CI = 0.835, 1.439) all produced positive effects, and intervention delivered by trained researchers, etc., produced significantly better results than parents and teachers (Q = 4.933, df = 1, *p* = 0.026). This suggests that there was a significant moderating effect of the intervener on the effect of the intervention. See Supplementary Figure S7 for details.

## 7. Discussion

The findings of this meta-analysis underscore the significant potential of information technology (IT)-based interventions in enhancing socio-emotional competence among individuals with ASD. The results revealed large effect sizes for emotion recognition (Hedges’ g = 0.805) and emotion understanding (Hedges’ g = 1.926), along with moderate effects for emotion expression (Hedges’ g = 0.711) and emotion regulation (Hedges’ g = 0.780). These outcomes align with prior research emphasizing the efficacy of IT tools in addressing core deficits in ASD (Rashidan et al. 2021; Weiss et al. 2018). Notably, the heterogeneity observed across studies highlights the importance of moderating factors, such as intervention technology and intervener expertise, in shaping intervention outcomes.

The superior performance of desktop-based interventions (Hedges’ g = 1.517) over mobile/wearable devices and emerging technologies (e.g., virtual reality) may stem from their reduced sensory demands and enhanced interactivity. Unlike immersive technologies, desktop tools minimize fatigue and isolation while allowing real-world social practice, which is critical for generalizing emotional skills (Lorenzo et al. 2016; Valentine et al. 2020). However, as noted in numerous desktop-based interventions studies, it often faces significant generalization challenges (Hopkins et al. 2011; Rice et al. 2015). Therefore, when implementing desktop-based interventions, practitioners should incorporate ecological bridging strategies—such as conducting in-session reviews of acquired knowledge, implementing self-monitoring protocols, and applying interventions in ecologically embedded settings—to connect therapeutic procedures with real-life contexts, thereby enhancing skill consolidation and generalization for ASD. Furthermore, interventions led by trained professionals (Hedges’ g = 1.137) outperformed those administered by parents or teachers, likely due to stricter adherence to protocols and technical expertise (Marino et al. 2019). This underscores the need for standardized training frameworks to optimize caregiver-led interventions.

The large effect sizes for emotion recognition and understanding may reflect the visual strengths of individuals with ASD, as IT tools leverage multisensory stimuli to enhance engagement and information processing (Golan et al. 2010). For instance, virtual reality systems that simulate real-world scenarios provide safe environments for repeated practice, thereby improving contextual emotion recognition (Ip et al. 2018). Similarly, emotion regulation improvements may be linked to IT tools’ ability to scaffold executive functioning through cognitive–behavioral strategies (Fage et al. 2019). These findings resonate with theories positing that enhanced cognitive flexibility and working memory can facilitate emotion regulation in ASD (Reyes et al. 2020).

Despite these promising results, limitations warrant consideration. First, the detected publication bias in emotion recognition studies may inflate effect estimates. Second, the small number of studies on emotion understanding (n = 3) and regulation (n = 4) limits generalizability. Third, heterogeneity in outcome measures and intervention designs complicates cross-study comparisons. Future research should prioritize standardized assessment tools, longitudinal follow-ups, and hybrid models integrating IT with in-person social training to enhance ecological validity. Additionally, personalized interventions tailored to individual cognitive profiles and socio-economic contexts are critical for addressing global disparities in ASD care.

## 8. Conclusions

In conclusion, this study substantiates IT-based interventions as potent tools for improving socio-emotional competencies in ASD, with desktop technologies and professionally delivered programs emerging as particularly effective. These insights not only inform clinical practice but also underscore the need for scalable, cost-effective solutions to expand access to underserved populations. Future work should explore adaptive algorithms, culturally sensitive designs, and multi-modal interventions to maximize therapeutic impact.

## Figures and Tables

**Table 1:** Results of risk quality assessment.

	Randomization Process	Deviations from Intended Interventions	Missing Outcome Data	Measurement of the Outcome	Selection of the Reported Result	Overall Bias
Beaumont et al. (2021)	Low	Low	Low	Low	Low	Low
Bekele et al. (2014)	Low	Low	Low	Low	Low	Low
Fage et al. (2019)	NA	Some concerns	High	Low	Low	High
Fridenson-Hayo et al. (2017)	NA	Low	Low	Low	Low	Low
Fridenson-Hayo et al. (2017)	Low	Low	Low	Low	Low	Low
Golan and Baron-Cohen (2006)	Low	Low	Low	Low	Low	Low
Golan and Baron-Cohen (2006)	Low	Low	Low	Low	Low	Low
Golan et al. (2010)	Low	Some concerns	Low	Low	Low	Some concerns
Herrero and Lorenzo (2020)	Low	High	Some concerns	Low	Low	High
Holeva et al. (2024)	Low	Low	Low	Low	Low	Low
Hopkins et al. (2011)	Low	Low	Some concerns	Low	Low	Some concerns
Ip et al. (2016)	Low	Some concerns	Low	Low	Low	Some concerns
Ip et al. (2018)	Some concerns	Some concerns	Low	Low	Low	Some concerns
Ip et al. (2022)	Low	Low	Low	Low	Low	Low
Kirst et al. (2022)	Low	Some concerns	Low	Low	Low	Some concerns
Marino et al. (2019)	Low	Low	Low	Low	Low	Low
Modugumudi et al. (2013)	Some concerns	Some concerns	Low	Low	Low	Some concerns
Rice et al. (2015)	Low	Low	Low	Low	Low	Low
Salvador et al. (2015)	NA	Some concerns	Low	Low	Low	Some concerns
Sosnowski et al. (2022)	Low	Low	Low	Low	Low	Low
Tang et al. (2021)	Low	Some concerns	Low	Low	Low	Some concerns
Thomeer et al. (2015)	Low	Some concerns	Low	Some concerns	Low	Some concerns
Vasilevska Petrovska and Trajkovski (2019)	Low	Low	Low	Low	Low	Low
Young and Posselt (2012)	Low	Low	Low	Low	Low	Low
Yuan and Ip (2018)	Some concerns	Some concerns	Low	Low	Low	Some concerns

**Table 2:** Results of the quality assessment of single-group pre- and post-test studies.

Study ID	Pre-Intervention		At Intervention	Post-Intervention				Overall Bias
	Bias Due to Confounding	Bias in Selection of Participants into the Study	Bias in Classification of Interventions	Bias Due to Deviations from Intended Interventions	Bias Due to Missing Data	Bias in Measurement of Outcomes	Bias in Selection of the Reported Result	
Didehbani et al. (2016)	Low	Low	Low	NA	Low	Low	Low	Low
Kandalaft et al. (2013)	Low	Low	Low	Low	Low	Moderate	Low	Moderate
Lin et al. (2023)	Serious	Low	Low	Low	Low	Low	Low	Serious
Serret et al. (2014)	Moderate	Low	Low	Low	Low	Low	Low	Moderate
Bazoolnejad et al. (2022)	Low	Low	Low	Low	Low	Low	Low	Low

## Data Availability

As a meta-analysis, all research data in this study have been presented in the article or supplementary materials, with no additional portions requiring provision.

## Supplementary Materials

The following supporting information can be downloaded at: https://www.mdpi.com/article/10.3390/jintelligence13080098/s1, Table S1: Characteristics of studies; Figure S1: Funnel plot-1:emotion recognition; Figure S2: Funnel plot-2:emotion understanding; Figure S3. Funnel plot-3:emotion expression; Figure S4. Funnel plot-4:emotion regulation; Figure S5. Forest plot-6: intervention duration; Figure S6. Forest plot-7: intervention frequency; Figure S7. Forest plot-8: intervention contexts; Figure S8. Forest plot-9: interveners.
