# Supplementary material for: Information Technology-Based Intervention on the Socio-Emotional Competence of Individuals with Autism Spectrum Disorders: A Systematic Review and Meta-Analysis
Source: J Intell. 2025 Aug 4;13(8):98. doi: 10.3390/jintelligence13080098 (PMC12387758; doi:10.3390/jintelligence13080098)
Supplement: Supplementary file 1 [file jintelligence-13-00098-s001.zip › jintelligence-3734696-supplementary.pdf]

Journal of Intelligence

MDPI

Supplementary Materials: Title Information Technology-Based Intervention on the Socio-Emotional Competence of Individuals with Autism Spectrum Disorders: A Systematic Review and Meta-Analysis

Yunshan Liu, Sirao Li, Yaping Huang and Dan Li *

School of Education, Hunan Normal University, Changsha 410081, China; 202230015014@hunnu.edu.cn (Y.L.); fancy@hunnu.edu.cn (S.L.); lys2yl@163.com (Y.H.)
* Correspondence: lidan@hunnu.edu.cn

Table S1. Characteristics of studies.

	Study design	Functional level	Sample Size (TG,CG)	Age(mean) (year)	Intervention category	Duration (Weeks)	Frequency /week	Context of intervention	Intervention Provider
**Bazoolnejad, 2022**	QED	high functioning	20(10, 10)	7-9	Emo Game software	4	3	NA	NA
**Beaumont, 2021**	RCT	IQ: Intervention: 100.45(13.34); Control:107.93(13.36)	70(35 ,35)	7-12	Video Gaming	10	10	home	parents
**Bekele, 2014**	RCT	IQ: Intervention: Male-120.1, (8.2), Female: 113.0, (12.0); Control:	20(10, 10)	13-17	Virtual reality	1 hour	NA	experiment room	parents

		Male:113.5(11.6), Female:107.5(2.5)							
**Didehbani, 2016**	Single-group pre- and post-tests	IQ:112.6(12.1)	30	7-16(11.4)	Virtual reality	5	2	school	Companions and doctors
**Fage, 2019**	RCT	IQ: Intervention: 69.07(30.64); Control: 60.53(19.61)	29(14, 15)	Intervention: 14.26(0.96); Control: 14.23 (1.28)	Emotion Regulation App	12	1	school classroom	assistants
**Fridenson-Hayo, 2017**	RCT	high functioning	43(23, 20)	6-9	Serious game	8	2 hours	home	parents
**Fridenson-Hayo,2017B**	RCT	high functioning	40(20,20)	6-9	Serious game	8	1	home	parents
**Golan, 2006**	RCT	IQ-Verbal: Intervention:108.3 (13.3) ; Control:109.7 (10.0)	65 (19,22)	Intervention: 30.5(10.3); Control: 30.9(11.2)	Software	10-15	2 hours	home	research worker
**Golan, 2006B**	RCT	IQ-Verbal: Intervention105.7 (16.1) ; Control:96.5 (15.5)	39(13,13)	Intervention: 25.5(9.3); Control:24.4(6.4)	Software and Tutor Group	10-15	1	home	research worker
**Golan, 2010**	RCT	IQ-Verbal: Intervention:98.3 (10.7) ; Control:99.4 (7.9)	56 (20:18:18)	4-7	Cartoon	4	7	home	research worker

**Herrero, 2020**	RCT	IQ:Low-High	14（7,7）	8-15	Virtual reality	NA	total：10 times	classroom	research worker
**Holeva, 2024**	RCT	IQ: Intervention: 81.37(13.09); Control: 84.74(11.87)	51 (25,26)	6-12	Robot	12	2	NA	therapists
**Hopkins, 2011**	RCT	IQ-Verbal: Intervention: 52.09(16.68); Control: 50.00(15.46)	25 (11,14)	6-10	Avatar Assistant(games)	6	2	school or after-school facility	research worker and teacher
**Hopkins, 2011B**	RCT	IQ-Verbal: Intervention: 92.05(18.63); Control: 93.09(21.91)	24 (13,11)	6-10	Avatar Assistant(games)	6	2	school or after-school facility	research worker and teacher
**Ip,2016**	RCT	IQ>70	32(16,16)	6-9（7）	Virtual Reality	14	2	-	trainer
**Ip,2018**	RCT	Raven: Interven-tion:94.9(17.49); Control:95.1(18.34)	94(47, 47)	108.4(18.75) months	Virtual reality	14	2	classroom	Trainers and assistants
**Ip,2022**	RCT	Non-verbal intelli-gence: Intervention:93(15.6); Control:92.5(16.5)	107(48,59)	Interven-tion: 101(19.9); Control: 112 (19.5) momths	Virtual reality	15	2	school	trainer and trained school-teacher
**Kandalaft, 2013**	Single-group pre- and post-tests	WASI:99-122	8	18-26	Virtual Reality	5	2	Social scenar-ios were con-structed	coach (lead cli-nician)

**Kirst,2022**	RCT	Verbal IQ : Intervention:97.6(17); Control:103(18)	82(42,40)	5 –10 years, 11 months	Serious game	6	>=2	home	Caregivers and experimenters
**Lin,2023**	Single-group pre- and post-tests	Nonverbal Intelligence:86-129	5	105months	Augmented reality storybook training modules	8	4	home	parents
**Marino,2020**	RCT	DQ: Intervention:95.9(13.1); Control:102(1.6)	14(7,7)	4–8	Robot	12	2	HomeLab.(an equipped room)	NA
**Modugumudi, 2013**	RCT	NA	20(10,10)	Intervention: 8-19 (11.6); Control: 7-19 (10.6)	Virtual Reality	24	NA	an equipped room	research worker
**Rice,2015**	RCT	IQ: Intervention:104.8(15.92); Control:98.53(12.43)	31(16,15)	5-11 (7.77)	Computer-assisted instruction	10	1	computer laboratory	teacher
**Salvador, 2015**	CT	high functioning autistic	22	7-13(9)	Zeno Robot	eight stages each lasting a maximum of three minutes	NA	the University of Denver	-
**Serret, 2014**	Single-group pre- and post-tests	WASI:70.5(27.6)	33	6-17(11.4)	Serious game	4	2	NA	a caregiver
**Sosnowski, 2022**	RCT	Verbal intelligence: Intervention:87.5(15.41); Control:86.33(19.30)	53(25,29)	4-14(8.56)	Applied Behavior Analysis with Gaze-Contingent Eye Trackin	6	5	NA	alone

**Tang,2021**	RCT	IQ: Intervention:110.30(14.66); Control:106.21(15.35)	25(11,14)	Intervention: 22.64(6.56); Control:25.11(6.37)	MindChip™(Computer-based)	10	1	laboratory	research worker
**Thomeer, 2015**	RCT	IQ: Intervention:101.59(15.34); Control:102.56(13.18)	43(22,21)	Intervention: 8.86(1.39); Control:8.57(1.16)	Mind Reading software	6	2	NA	psychologist
**Vasilevska Petrovska, 2019**	RCT	59%having intellectual disability; 41% had mild ID	34(17,16)	7-15(10.91)	Computer-Based Intervention	8	2	home	research worker
**Young, 2012**	RCT	Nonverbal IQ: Intervention: 11.31(4.17); Control: 8.67(4.05)	25(13,12)	4-8	The Transporters DVD	3	at least three episodes a day	home	parents
**Yuan,2018**	RCT	-	72(36,36)	Intervention:107.6(13.27); Control:104.8(13.83)	Virtual reality	NA	NA	NA	trainer

Note: This table includes only the first author's name; distinct findings from the same study are denoted by capital letters; Intervention targets: a = emotion recognition, b = emotion understanding, c = emotion expression, d = emotion regulation.

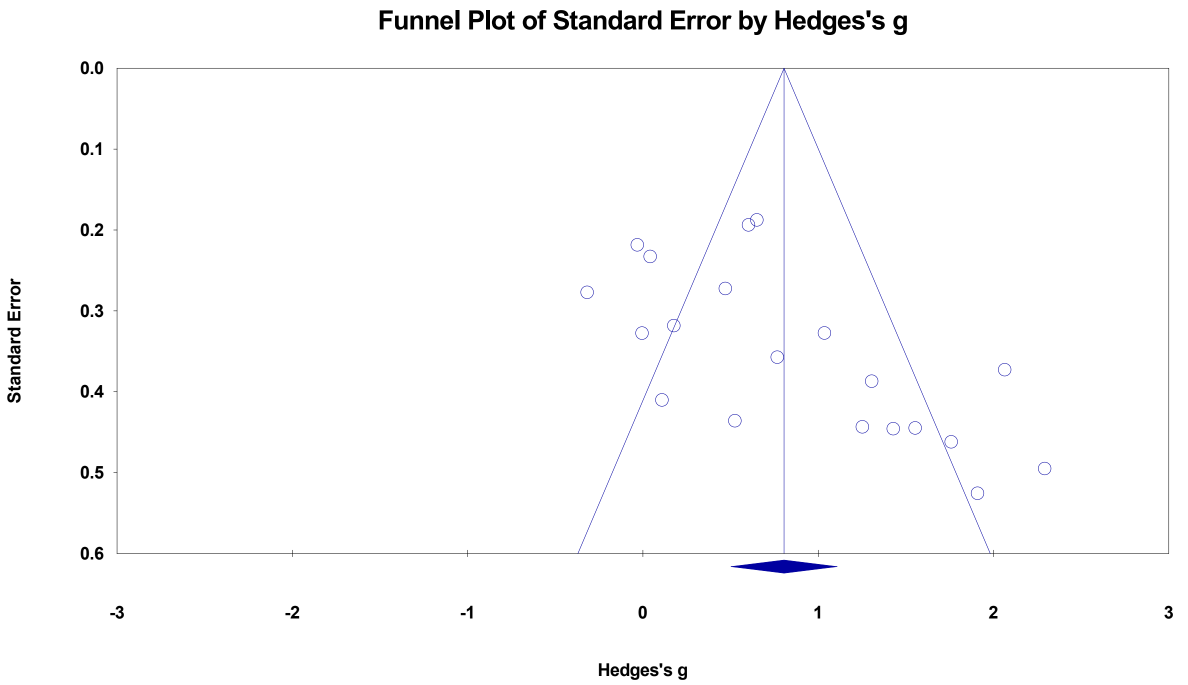

Figure S1. Funnel plot-1:emotion recognition.

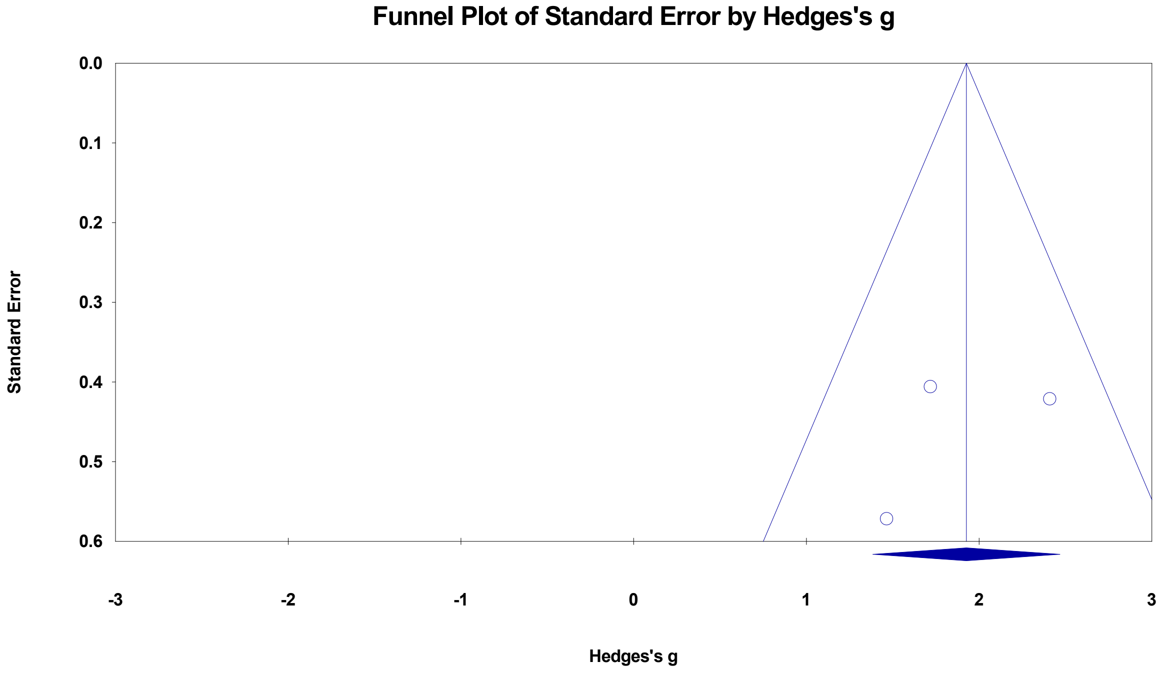

Figure S2. Funnel plot-2:emotion understanding.

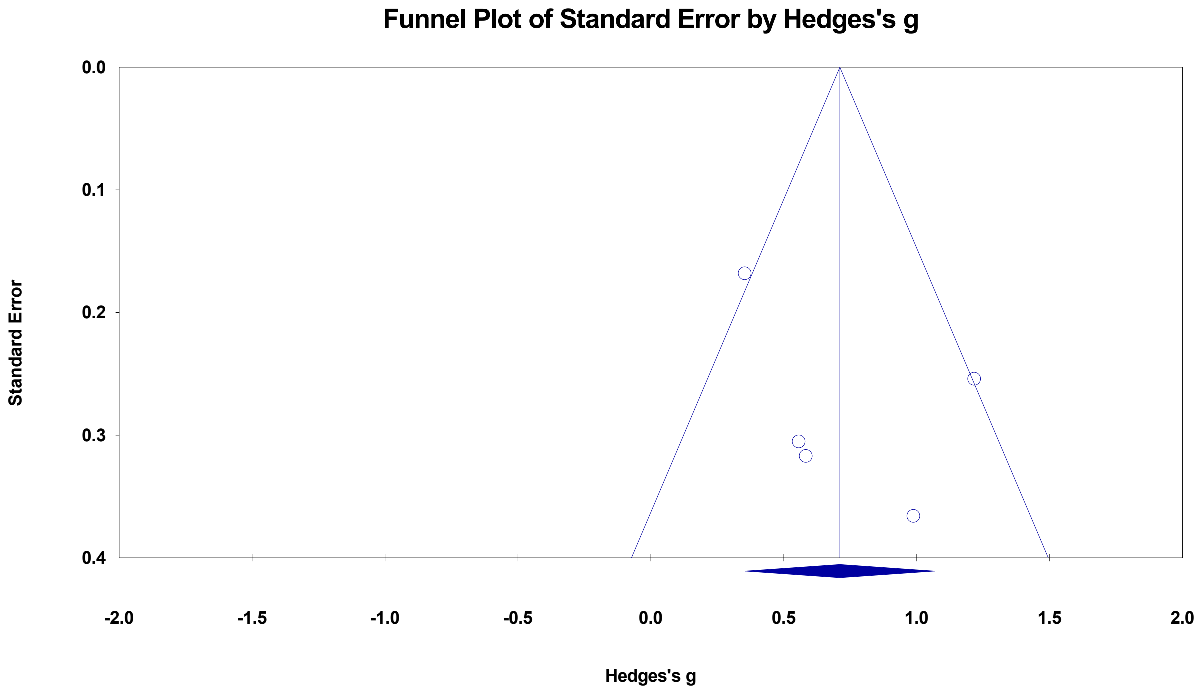

Figure S3. Funnel plot-3:emotion expression.

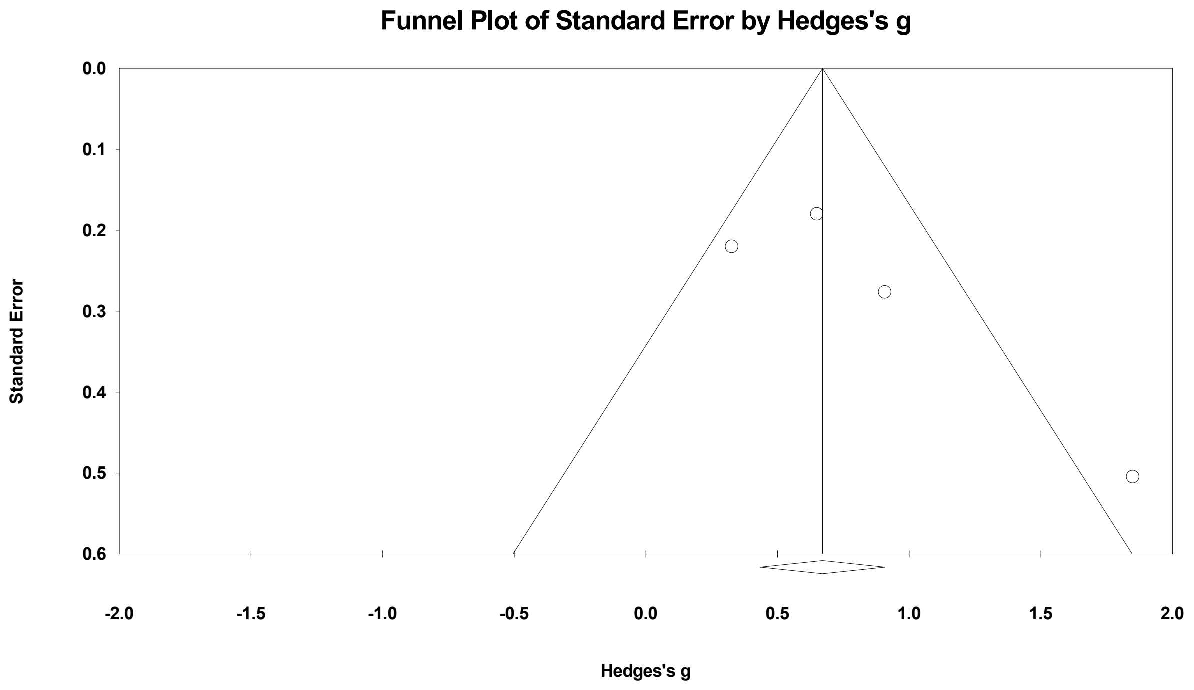

Figure S4. Funnel plot-4:emotion regulation.

Group by time	Study name	Hedges's g	Standard error	Variance	Lower limit	Upper limit	Z-Value	p-Value
		**Statistics for each study**						
1.00	Bazoolnejad,2022	1.850	0.505	0.255	0.861	2.840	3.666	0.000
1.00	Bekele,2014	0.527	0.436	0.190	-0.328	1.382	1.207	0.227
1.00	Didehbani,2016	0.605	0.194	0.038	0.224	0.985	3.113	0.002
1.00	Golan,2010	2.411	0.421	0.178	1.585	3.237	5.720	0.000
1.00	Hopkins,2011	1.761	0.462	0.214	0.855	2.667	3.808	0.000
1.00	Hopkins,2011B	1.430	0.446	0.199	0.556	2.305	3.206	0.001
1.00	Kandalaft,2013	1.254	0.444	0.197	0.384	2.124	2.825	0.005
1.00	Kirst,2022	-0.030	0.219	0.048	-0.459	0.399	-0.136	0.892
1.00	Kirst,2022B	0.327	0.220	0.049	-0.105	0.759	1.484	0.138
1.00	Serret,2014	0.652	0.188	0.035	0.284	1.021	3.471	0.001
1.00	Sosnowski,2022	0.472	0.273	0.074	-0.062	1.007	1.731	0.083
1.00	Thomeer,2015	2.066	0.373	0.139	1.335	2.798	5.536	0.000
1.00	Thomeer,2015B	0.558	0.305	0.093	-0.041	1.156	1.826	0.068
1.00	Young,2012	1.555	0.445	0.198	0.683	2.428	3.493	0.000
1.00		0.746	0.079	0.006	0.592	0.900	9.499	0.000
2.00	Beaumont,2021	0.908	0.277	0.077	0.366	1.451	3.283	0.001
2.00	Fridenson-Hayo,2017	0.178	0.319	0.102	-0.446	0.803	0.560	0.576
2.00	Fridenson-Hayo,2017B	-0.003	0.328	0.108	-0.646	0.640	-0.009	0.993
2.00	Golan,2006	1.038	0.328	0.107	0.396	1.681	3.167	0.002
2.00	Golan,2006B	2.294	0.495	0.246	1.323	3.265	4.630	0.000
2.00	Holeva,2024	-0.316	0.278	0.077	-0.860	0.228	-1.138	0.255
2.00	Ip,2016	0.769	0.358	0.128	0.067	1.470	2.148	0.032
2.00	Ip,2016B	0.989	0.366	0.134	0.271	1.707	2.701	0.007
2.00	Ip,2018	0.044	0.233	0.054	-0.413	0.501	0.188	0.851
2.00	Ip,2018B	1.218	0.254	0.065	0.719	1.716	4.789	0.000
2.00	Ip,2022	0.980	0.204	0.042	0.580	1.381	4.799	0.000
2.00	Marino,2020	1.466	0.572	0.327	0.345	2.587	2.563	0.010
2.00	Modugumudi,2013	0.584	0.317	0.101	-0.038	1.206	1.841	0.066
2.00	Rice,2015	1.308	0.387	0.150	0.548	2.067	3.375	0.001
2.00	Tang,2021	1.911	0.526	0.277	0.880	2.942	3.634	0.000
2.00	Vasilevska Petrovska,2019	1.720	0.406	0.165	0.924	2.516	4.234	0.000
2.00		0.754	0.079	0.006	0.599	0.908	9.564	0.000
Overall		0.750	0.056	0.003	0.641	0.859	13.479	0.000

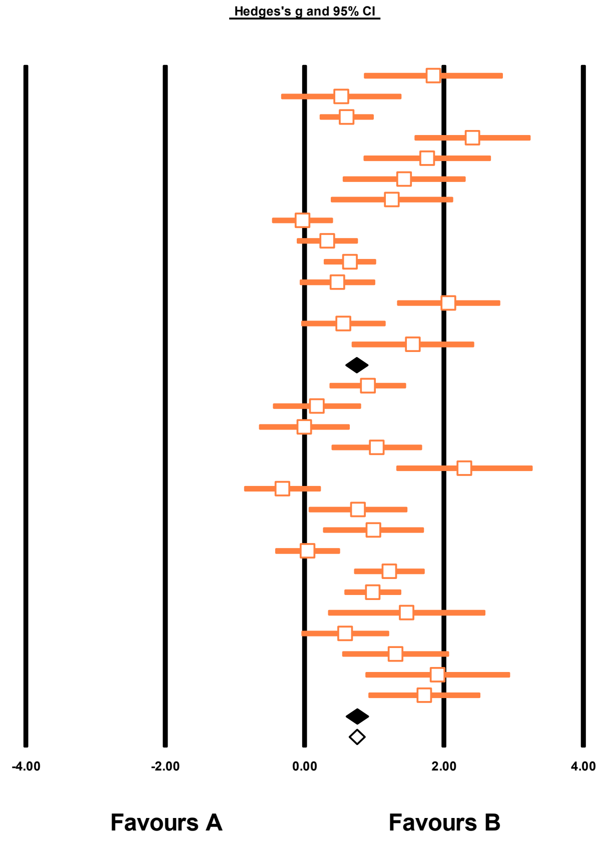

Meta Analysis

Figure S5. Forest plot-6: intervention duration.

Group by Intervention frequency	Study name	Hedges's g	Standard error	Variance	Lower limit	Upper limit	Z-Value	p-Value
		Statistics for each study						
1.00	Didehbani,2016	0.605	0.194	0.038	0.224	0.985	3.113	0.002
1.00	Fridenson-Hayo,2017	0.178	0.319	0.102	-0.446	0.803	0.560	0.576
1.00	Fridenson-Hayo,2017B	-0.003	0.328	0.108	-0.646	0.640	-0.009	0.993
1.00	Golan,2006	1.038	0.328	0.107	0.396	1.681	3.167	0.002
1.00	Golan,2006B	2.294	0.495	0.246	1.323	3.265	4.630	0.000
1.00	Holeva,2024	-0.316	0.278	0.077	-0.860	0.228	-1.138	0.255
1.00	Hopkins,2011	1.761	0.462	0.214	0.855	2.667	3.808	0.000
1.00	Hopkins,2011B	1.430	0.446	0.199	0.556	2.305	3.206	0.001
1.00	Ip,2016	0.769	0.358	0.128	0.067	1.470	2.148	0.032
1.00	Ip,2016B	0.989	0.366	0.134	0.271	1.707	2.701	0.007
1.00	Ip,2018	0.044	0.233	0.054	-0.413	0.501	0.188	0.851
1.00	Ip,2018B	1.218	0.254	0.065	0.719	1.716	4.789	0.000
1.00	Ip,2022	0.980	0.204	0.042	0.580	1.381	4.799	0.000
1.00	Kandalaft,2013	1.254	0.444	0.197	0.384	2.124	2.825	0.005
1.00	Marino,2020	1.466	0.572	0.327	0.345	2.587	2.563	0.010
1.00	Rice,2015	1.308	0.387	0.150	0.548	2.067	3.375	0.001
1.00	Serret,2014	0.652	0.188	0.035	0.284	1.021	3.471	0.001
1.00	Tang,2021	1.911	0.526	0.277	0.880	2.942	3.634	0.000
1.00	Thomeer,2015	2.066	0.373	0.139	1.335	2.798	5.536	0.000
1.00	Thomeer,2015B	0.558	0.305	0.093	-0.041	1.156	1.826	0.068
1.00	Vasilevska Petrovska,2019	1.720	0.406	0.165	0.924	2.516	4.234	0.000
1.00		0.802	0.067	0.004	0.671	0.932	12.038	0.000
2.00	Bazoolnejad,2022	1.850	0.505	0.255	0.861	2.840	3.666	0.000
2.00	Beaumont,2021	0.908	0.277	0.077	0.366	1.451	3.283	0.001
2.00	Golan,2010	2.411	0.421	0.178	1.585	3.237	5.720	0.000
2.00	Kirst,2022	-0.030	0.219	0.048	-0.459	0.399	-0.136	0.892
2.00	Kirst,2022B	0.327	0.220	0.049	-0.105	0.759	1.484	0.138
2.00	Sosnowski,2022	0.472	0.273	0.074	-0.062	1.007	1.731	0.083
2.00	Young,2012	1.555	0.445	0.198	0.683	2.428	3.493	0.000
2.00		0.642	0.110	0.012	0.426	0.858	5.833	0.000
Overall		0.759	0.057	0.003	0.647	0.871	13.319	0.000

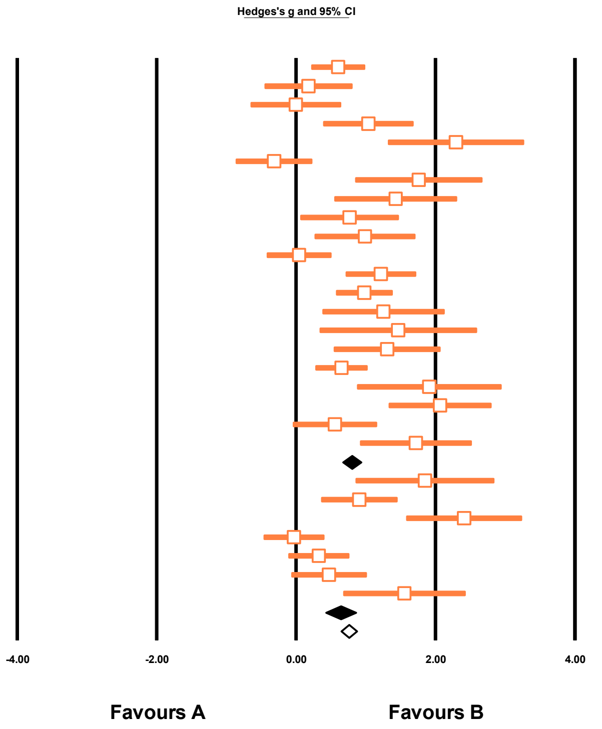

Meta Analysis

Figure S6. Forest plot-7: intervention frequency.

Group by Place	Study name	Hedges's g	Standard error	Variance	Lower limit	Upper limit	Z-Value	p-Value
1.00	Beaumont,2021	0.908	0.277	0.077	0.366	1.451	3.283	0.001
1.00	Didehbani,2016	0.605	0.194	0.038	0.224	0.985	3.113	0.002
1.00	Fridenson-Hayo,2017	0.178	0.319	0.102	-0.446	0.803	0.560	0.576
1.00	Fridenson-Hayo,2017B	-0.003	0.328	0.108	-0.646	0.640	-0.009	0.993
1.00	Golan,2006	1.038	0.328	0.107	0.396	1.681	3.167	0.002
1.00	Golan,2006B	2.294	0.495	0.246	1.323	3.265	4.630	0.000
1.00	Golan,2010	2.411	0.421	0.178	1.585	3.237	5.720	0.000
1.00	Hopkins,2011	1.761	0.462	0.214	0.855	2.667	3.808	0.000
1.00	Hopkins,2011B	1.430	0.446	0.199	0.556	2.305	3.206	0.001
1.00	Ip,2018	0.044	0.233	0.054	-0.413	0.501	0.188	0.851
1.00	Ip,2018B	1.218	0.254	0.065	0.719	1.716	4.789	0.000
1.00	Ip,2022	0.980	0.204	0.042	0.580	1.381	4.799	0.000
1.00	Kirst,2022	-0.030	0.219	0.048	-0.459	0.399	-0.136	0.892
1.00	Kirst,2022B	0.327	0.220	0.049	-0.105	0.759	1.484	0.138
1.00	Vasilevska Petrovska,2019	1.720	0.406	0.165	0.924	2.516	4.234	0.000
1.00	Young,2012	1.555	0.445	0.198	0.683	2.428	3.493	0.000
1.00		0.734	0.071	0.005	0.594	0.873	10.319	0.000
2.00	Bekele,2014	0.527	0.436	0.190	-0.328	1.382	1.207	0.227
2.00	Kandalaft,2013	1.254	0.444	0.197	0.384	2.124	2.825	0.005
2.00	Marino,2020	1.466	0.572	0.327	0.345	2.587	2.563	0.010
2.00	Modugumudi,2013	0.584	0.317	0.101	-0.038	1.206	1.841	0.066
2.00	Rice,2015	1.308	0.387	0.150	0.548	2.067	3.375	0.001
2.00	Salvador,2015	0.111	0.411	0.169	-0.694	0.915	0.270	0.787
2.00	Tang,2021	1.911	0.526	0.277	0.880	2.942	3.634	0.000
2.00		0.903	0.159	0.025	0.591	1.215	5.677	0.000
Overall		0.762	0.065	0.004	0.635	0.889	11.737	0.000

Hedges's g and 95% CI

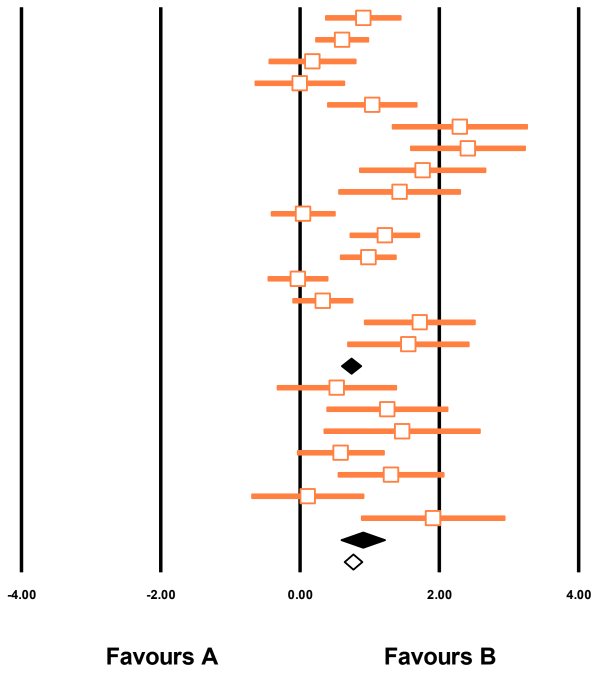

Meta Analysis

Figure S7. Forest plot-8: intervention contexts.

Group by Interveners	Study name	Hedges's g	Standard error	Variance	Lower limit	Upper limit	Z-Value	p-Value
		Statistics for each study						
1.00	Beaumont,2021	0.908	0.277	0.077	0.366	1.451	3.283	0.001
1.00	Bekele,2014	0.527	0.436	0.190	-0.328	1.382	1.207	0.227
1.00	Fridenson-Hayo,2017	0.178	0.319	0.102	-0.446	0.803	0.560	0.576
1.00	Fridenson-Hayo,2017B	-0.003	0.328	0.108	-0.646	0.640	-0.009	0.993
1.00	Holeva,2024	-0.316	0.278	0.077	-0.860	0.228	-1.138	0.255
1.00	Rice,2015	1.308	0.387	0.150	0.548	2.067	3.375	0.001
1.00	Serret,2014	0.652	0.188	0.035	0.284	1.021	3.471	0.001
1.00	Young,2012	1.555	0.445	0.198	0.683	2.428	3.493	0.000
1.00		0.523	0.105	0.011	0.317	0.729	4.972	0.000
2.00	Didehbani,2016	0.605	0.194	0.038	0.224	0.985	3.113	0.002
2.00	Golan,2006	1.038	0.328	0.107	0.396	1.681	3.167	0.002
2.00	Golan,2006B	2.294	0.495	0.246	1.323	3.265	4.630	0.000
2.00	Golan,2010	2.411	0.421	0.178	1.585	3.237	5.720	0.000
2.00	Hopkins,2011	1.761	0.462	0.214	0.855	2.667	3.808	0.000
2.00	Hopkins,2011B	1.430	0.446	0.199	0.556	2.305	3.206	0.001
2.00	Ip,2016	0.769	0.358	0.128	0.067	1.470	2.148	0.032
2.00	Ip,2016B	0.989	0.366	0.134	0.271	1.707	2.701	0.007
2.00	Ip,2018	0.044	0.233	0.054	-0.413	0.501	0.188	0.851
2.00	Ip,2018B	1.218	0.254	0.065	0.719	1.716	4.789	0.000
2.00	Ip,2022	0.980	0.204	0.042	0.580	1.381	4.799	0.000
2.00	Kandalaft,2013	1.254	0.444	0.197	0.384	2.124	2.825	0.005
2.00	Modugumudi,2013	0.584	0.317	0.101	-0.038	1.206	1.841	0.066
2.00	Tang,2021	1.911	0.526	0.277	0.880	2.942	3.634	0.000
2.00	Thomeer,2015	2.066	0.373	0.139	1.335	2.798	5.536	0.000
2.00	Thomeer,2015B	0.558	0.305	0.093	-0.041	1.156	1.826	0.068
2.00	Vasilevska Petrovska,2019	1.720	0.406	0.165	0.924	2.516	4.234	0.000
2.00	Yuan,2018	0.355	0.168	0.028	0.025	0.684	2.106	0.035
2.00		0.889	0.070	0.005	0.753	1.026	12.769	0.000
Overall		0.778	0.058	0.003	0.664	0.891	13.392	0.000

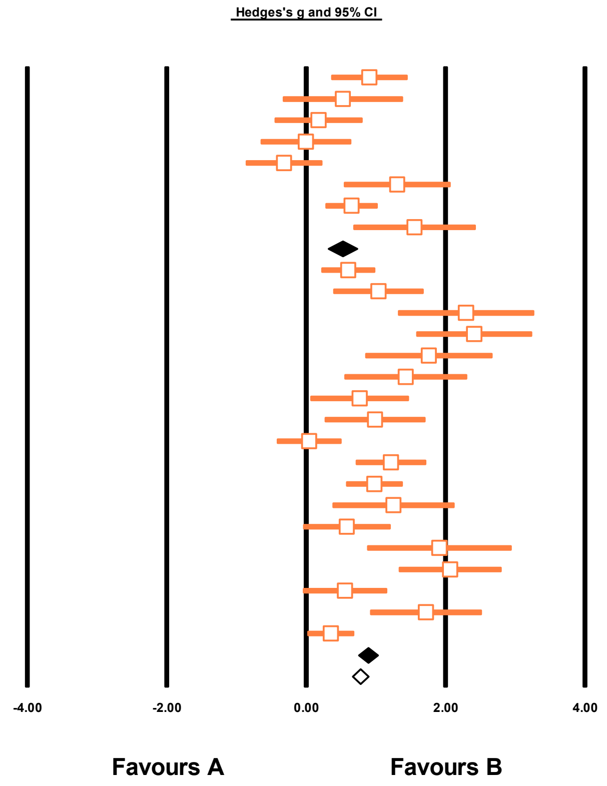

Meta Analysis

Figure S8. Forest plot-9: interveners.
